# Brain Abscesses in Crohn’s Disease as a Complication of Infliximab Therapy

**DOI:** 10.7759/cureus.15449

**Published:** 2021-06-04

**Authors:** Mahmoud M Mansour, Muhammad Mubarak, Harleen Chela, Yezaz A Ghouri

**Affiliations:** 1 Internal Medicine, University of Missouri School of Medicine, Columbia, USA; 2 Internal Medicine/Gastroenterology and Hepatology, University of Missouri School of Medicine, Columbia, USA

**Keywords:** infliximab, streptococcus constellatus, brain abscess, crohn’s disease, inflammatory bowel disease, anti-tnf-α

## Abstract

Infliximab therapy is highly effective in the treatment of Crohn’s disease. Infliximab-induced immunosuppression increases the risk for various infections, including opportunistic infections. We describe a case of brain abscesses as a complication of infliximab therapy in a 65-year-old man. It was elucidated that the brain abscesses developed from the presumed hematogenous spread of bacteria from recently treated paraspinal abscesses. Close attention should be given to patients on infliximab therapy presenting with any neurological sign or symptom to allow for timely diagnosis and prompt management of a potential central nervous system infection.

## Introduction

Infliximab is a chimeric monoclonal antibody against the cytokine tumor necrosis factor-alpha (TNF-α) and is used to treat a wide variety of autoimmune and inflammatory diseases, such as Crohn’s disease (CD), ulcerative colitis, rheumatoid arthritis, and psoriatic arthritis [1]. Infection-related complications of TNF-α inhibitors are well described in the literature. Traditionally, TNF-α inhibitors have been associated with infections such as tuberculosis, hepatitis B, listeria, fungi, and viruses [2-4]. However, there is an increasing number of reports investigating TNF-α inhibitor-associated bacterial infections involving multiple organ systems, including soft tissue, muscle, heart, and liver [3,5]. In this case report, we describe brain abscesses, presumed to be secondary to S*treptococcus constellatus* bacteremia, that developed secondary to the hematogenous spread of bacteria from previously treated paraspinal abscesses in a patient on infliximab therapy for CD.

## Case presentation

A 65-year-old male with a past medical history of stricturing and fistulizing CD, who had been medically managed with infliximab therapy for two years, presented to the emergency department with left upper extremity weakness and left-sided facial droop. This presentation occurred one week after completing treatment for paraspinal abscesses that grew methicillin-sensitive *Staphylococcus aureus*, *Escherichia coli*, and *S. constellatus*, which was associated with *S. constellatus* bacteremia. At that time, infliximab therapy was held, and complete resolution of the paraspinal abscesses was achieved with surgical drainage and three weeks of piperacillin-tazobactam therapy.

**Figure 1:**
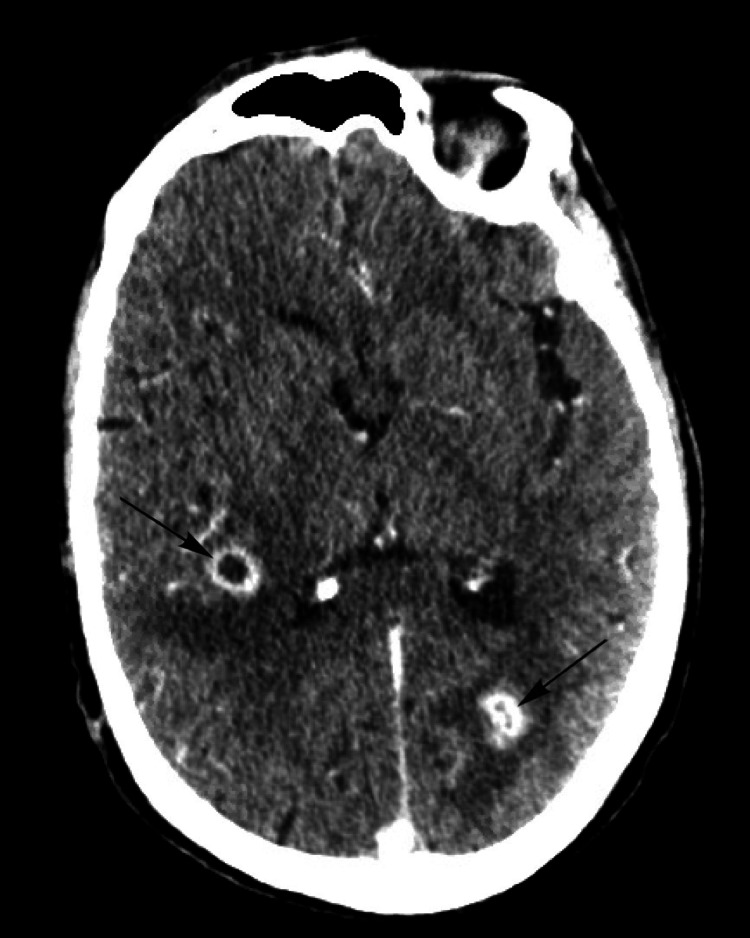
CT head with intravenous contrast showing two rim-enhancing lesions (arrows) in the right temporal and left parietal lobes with surrounding vasogenic edema. CT: computed tomography

The patient’s current presentation with focal neurological symptoms was concerning for a cerebrovascular accident. Computed tomography (CT) scan of the head without intravenous (IV) contrast demonstrated multifocal areas of vasogenic edema within the left parieto-occipital, right superior temporal, and the right superior frontal lobes. Subsequent CT imaging with IV contrast revealed two rim-enhancing lesions with surrounding vasogenic edema in the right temporal and left inferior parietal lobule measuring 1.4 cm and 1.3 cm, respectively (Figure 1). Whole-body positron emission tomography/computed tomography demonstrated increased fluorodeoxyglucose avidity by multiple perihilar and mediastinal lymph nodes. Mediastinal lymph node biopsy through endoscopic bronchial ultrasound was negative for malignant cells. Infectious workup with blood cultures and lumbar puncture was unrevealing.

Considering rim-enhancing intracranial lesions and the absence of obvious infarcted cerebral territory or malignant etiology, cerebral abscesses were thought to describe the patient’s presentation.

**Figure 2:**
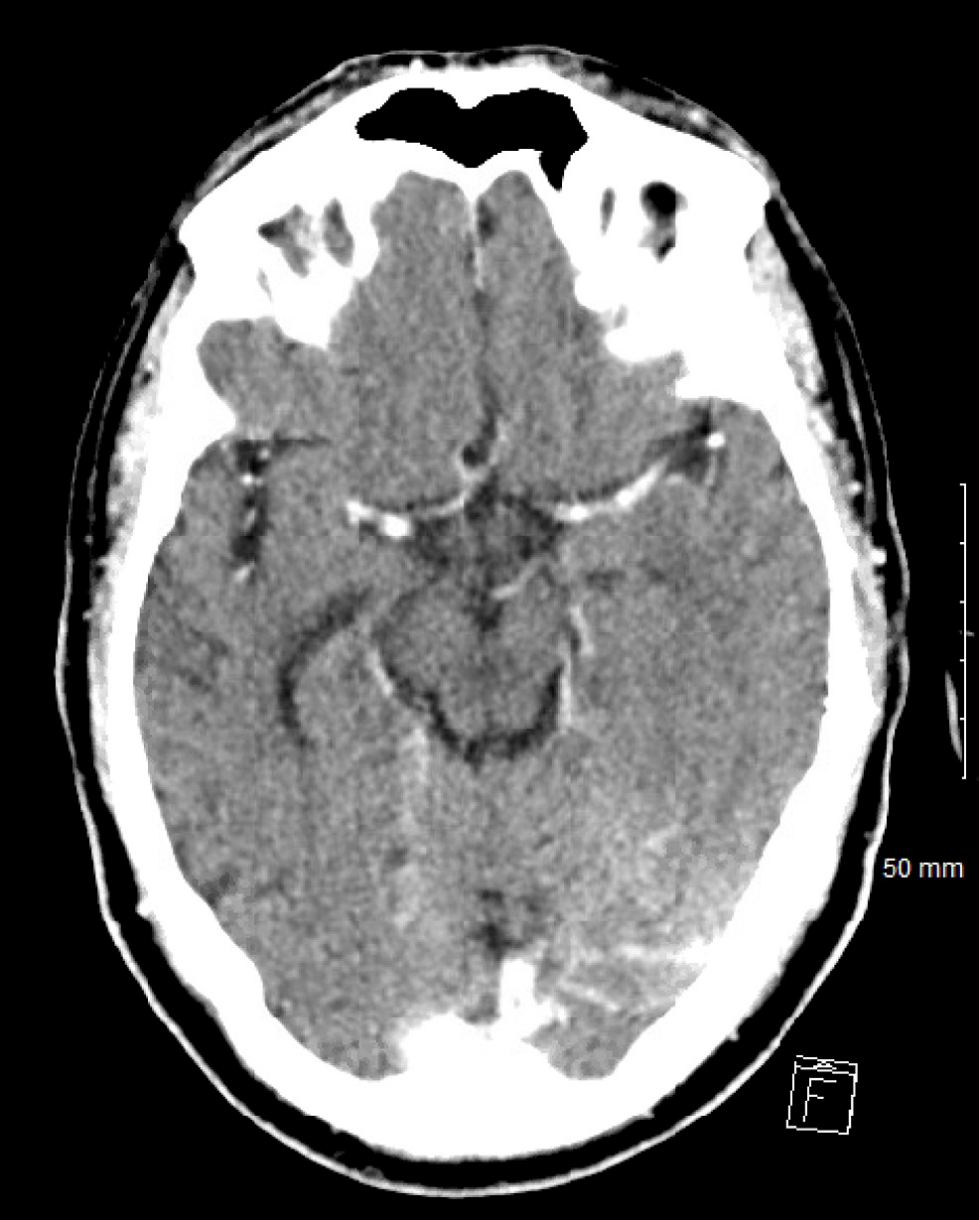
CT head with intravenous contrast showing complete resolution of the intracerebral abscesses following treatment with ceftriaxone. CT: computed tomography

Due to the inaccessibility of the brain lesions, aspiration biopsy was not performed. After reviewing the culture results from the last admission, the infectious disease experts recommended that the patient be started on IV ceftriaxone treatment for brain abscesses due to presumed hematogenous spread of *S. constellatus* during the period of bacteremia four weeks prior. Over an eight-week IV ceftriaxone course, serial CT images demonstrated continuous improvement of abscesses. Following the completion of antimicrobial therapy, the patient had total radiographic (Figure 2) and neurological deficit resolution. For further CD treatment, the patient was switched to vedolizumab for its gut-selective mechanism of action, thus sparing him from systemic immunosuppression.

## Discussion

Serious bacterial infections, leading to hospitalization or death, have been described in patients on TNF-α inhibitor therapy in multiple observational studies [2,3]. Given the central role of TNF-α in the human immune response, TNF-α inhibitors, such as infliximab, diminishes both humoral and cellular immunity by suppressing T-cell proinflammatory response, leaving patients vulnerable to various infections [1]. Pulmonary, skin, and soft tissue infections are among the most common serious infections encountered in patients on TNF-α inhibitor therapy [3].

*S. constellatus* is a gram-positive cocci belonging to the *S. anginosus* group, a part of the gastrointestinal and urogenital flora [6]. *S. constellatus* is an uncommon cause of soft tissue abscesses and a rare cause of brain abscesses. However, immunosuppressed states increase the risk of developing invasive pyogenic infections due to *S. constellatus* [7,8].

The current case discusses brain abscesses presumed to be caused by *S. constellatus* in a patient with CD on infliximab therapy. After ruling out other etiologies, including cerebrovascular accident and malignancy, brain abscesses were thought to cause the findings found on brain CT. The infectious disease experts hypothesized that the hematogenous spread of *S. constellatus* to the central nervous system (CNS) ensued at the time of paraspinal infection. Although piperacillin-tazobactam therapy successfully targeted the bacteria that grew in the paraspinal abscesses and blood, brain abscesses were thought to have developed due to the poor ability of piperacillin-tazobactam to penetrate the blood-brain barrier [9].

Needle aspiration is generally the preferred initial treatment of choice for brain abscesses [10]. Cerebrospinal fluid (CSF) findings are usually nonspecific, and CSF cultures are rarely positive in patients with brain abscesses [11]. In our patient, lumbar puncture was inconclusive, and brain abscesses were inaccessible to aspiration. Despite the lack of bacteriologic diagnosis, successful empiric management with eight weeks of ceftriaxone, which readily penetrates the blood-brain barrier, confirmed the diagnosis of brain abscesses.

## Conclusions

We present a case of brain abscesses in an individual on infliximab therapy for underlying CD. The brain abscesses were hypothesized to have developed after hematogenous spread of bacteria from recently treated paraspinal abscesses. Complete radiographic resolution with ceftriaxone therapy confirmed the diagnosis of brain abscesses that were presumed to be secondary to *S. constellatus*. This case highlights the need to be vigilant for CNS infections among patients on infliximab therapy presenting with neurological deficits. Recognition and prompt management are vital in preventing permanent neurological injury and death.

## References

[1] Her M, Kavanaugh A (2016). Alterations in immune function with biologic therapies for autoimmune disease. J Allergy Clin Immunol.

[2] Baddley JW, Winthrop KL, Chen L (2014). Non-viral opportunistic infections in new users of tumour necrosis factor inhibitor therapy: results of the SAfety Assessment of Biologic ThERapy (SABER) study. Ann Rheum Dis.

[3] Dixon WG, Watson K, Lunt M, Hyrich KL, Silman AJ, Symmons DP (2006). Rates of serious infection, including site-specific and bacterial intracellular infection, in rheumatoid arthritis patients receiving anti-tumor necrosis factor therapy: results from the British Society for Rheumatology Biologics Register. Arthritis Rheum.

[4] Crum NF, Lederman ER, Wallace MR (2005). Infections associated with tumor necrosis factor-alpha antagonists. Medicine (Baltimore).

[5] Roos JC, Ostor AJ (2006). Orbital cellulitis in a patient receiving infliximab for ankylosing spondylitis. Am J Ophthalmol.

[6] Claridge JE 3rd, Attorri S, Musher DM, Hebert J, Dunbar S (2001). Streptococcus intermedius, Streptococcus constellatus, and Streptococcus anginosus ("Streptococcus milleri group") are of different clinical importance and are not equally associated with abscess. Clin Infect Dis.

[7] Kragha KO (2016). Multiple brain abscesses due to Streptococcus anginosus: prediction of mortality by an imaging severity index score. Case Rep Radiol.

[8] Şenol Ö, Süslü HT, Tatarlı N, Tiryaki M, Güçlü B (2016). Thalamic abscess caused by a rare pathogen: Streptococcus constellatus. Pan Afr Med J.

[9] Nau R, Kinzig-Schippers M, Sörgel F (1997). Kinetics of piperacillin and tazobactam in ventricular cerebrospinal fluid of hydrocephalic patients. Antimicrob Agents Chemother.

[10] Ratnaike TE, Das S, Gregson BA, Mendelow AD (2011). A review of brain abscess surgical treatment--78 years: aspiration versus excision. World Neurosurg.

[11] Mathisen GE, Johnson JP (1997). Brain abscess. Clin Infect Dis.

